# Lung Volume, Breathing Pattern and Ventilation Inhomogeneity in Preterm and Term Infants

**DOI:** 10.1371/journal.pone.0004635

**Published:** 2009-02-27

**Authors:** Philipp Latzin, Stefan Roth, Cindy Thamrin, Gerard J. Hutten, Isabelle Pramana, Claudia E. Kuehni, Carmen Casaulta, Matthias Nelle, Thomas Riedel, Urs Frey

**Affiliations:** 1 Division of Respiratory Medicine, Department of Paediatrics, Inselspital and University of Bern, Bern, Switzerland; 2 Institute of Social and Preventive Medicine (ISPM), University of Bern, Bern, Switzerland; 3 Division of Paediatric and Neonatal Intensive Care, Department of Paediatrics, Inselspital and University of Bern, Bern, Switzerland; Helmholtz Zentrum München/Ludwig-Maximilians-University Munich, Germany

## Abstract

**Background:**

Morphological changes in preterm infants with bronchopulmonary dysplasia (BPD) have functional consequences on lung volume, ventilation inhomogeneity and respiratory mechanics. Although some studies have shown lower lung volumes and increased ventilation inhomogeneity in BPD infants, conflicting results exist possibly due to differences in sedation and measurement techniques.

**Methodology/Principal Findings:**

We studied 127 infants with BPD, 58 preterm infants without BPD and 239 healthy term-born infants, at a matched post-conceptional age of 44 weeks during quiet natural sleep according to ATS/ERS standards. Lung function parameters measured were functional residual capacity (FRC) and ventilation inhomogeneity by multiple breath washout as well as tidal breathing parameters. Preterm infants with BPD had only marginally lower FRC (21.4 mL/kg) than preterm infants without BPD (23.4 mL/kg) and term-born infants (22.6 mL/kg), though there was no trend with disease severity. They also showed higher respiratory rates and lower ratios of time to peak expiratory flow and expiratory time (*t*_PTEF_/*t*_E_) than healthy preterm and term controls. These changes were related to disease severity. No differences were found for ventilation inhomogeneity.

**Conclusions:**

Our results suggest that preterm infants with BPD have a high capacity to maintain functional lung volume during natural sleep. The alterations in breathing pattern with disease severity may reflect presence of adaptive mechanisms to cope with the disease process.

## Introduction

Chronic lung disease of infancy remains one of the major complications in premature infants, with possible impact upon short- and long-term respiratory morbidity [1]–[3]. Lung development in preterm infants is interrupted during the saccular phase of the normal maturational process usually taking place *in utero*. This leads to disturbed alveolar septation with fewer but larger alveoli and impaired vascular growth in these infants [4], [5]. The immaturity of the lungs together with other factors such as inflammation, disturbed vascularisation, hyperoxia and volu- or barotrauma during treatment may lead to the clinical picture of bronchopulmonary dysplasia (BPD) [4], [6].

These morphological changes in BPD lungs have functional consequences on lung volume, ventilation homogeneity and mechanics of the respiratory system, as reviewed recently [7]–[10]. End-expiratory lung volume (functional residual capacity, FRC) has been shown to be diminished in healthy preterm and BPD infants [11], [12]. Furthermore, these infants have increased ventilation inhomogeneity [11], [12] as well as reduced forced expiratory flows, without catching up from this diminished airway growth within the first two years of life [13]–[16]. On the other hand, there are also reports of comparable lung volumes in infants with BPD, preterm and term-born controls [17], [18]. It is difficult to draw clear conclusions from these studies, partly because of disregard of possible changes in breathing pattern, e.g. due to sedation, test gas used [19], [20] or sighs [21]. Furthermore, the use of 100% oxygen in the nitrogen washout test used in many studies to determine lung volume may induce atelectasis [22], and the effect may be more pronounced in young and preterm infants [23]. Differences in measurement techniques [24] or inappropriate matching for age, weight and length of the control groups [15], [17] also limit physiological implications of these studies.

To obtain valid information on the natural and dynamic pattern of breathing and overcome factors potentially causing the above mentioned inconsistencies, it is necessary to measure tidal breathing parameters in addition to lung volume and ventilation inhomogeneity in a large sample of unsedated infants, matching preterm infants with contemporarily recruited healthy controls, and using the same appropriate methods for all infants adhering to recently published ATS/ERS standards for infant lung function testing [25].

With this in mind, the aim of our study was to compare lung volume, ventilation inhomogeneity and tidal breathing parameters in preterm infants with and without BPD with term-born infants matched for post-conceptional age at study date. In addition, we aimed to assess which clinical factors are associated with the respective lung function parameters in BPD infants.

## Materials and Methods

### Study design

In this cross-sectional observational study, we measured lung function in preterm infants with and without BPD and healthy term-born infants. Subjects were matched for a post-conceptional age of 44 weeks at study date and recruited during 1999–2007 within the region of Bern, Switzerland.

#### Ethics Statement

The Bernese Cantonal Ethics Committee approved the study protocol and written informed consent was obtained from parents.

#### Preterm infants

Preterm infants of less than 37 weeks gestational age hospitalized at the University Children's hospital of Bern, Switzerland, were recruited during initial hospital stay. Detailed prenatal and neonatal history was routinely recorded during the hospital stay into a database. Any missing parameters were supplemented at the time of lung function or by retrospective chart analysis.

Based on the ATS definition of BPD, we categorized preterm infants into healthy and mild, moderate and severe BPD, respectively [6]. BPD was defined as supplemental oxygen requirement for at least 28 days. Time point of assessment for BPD severity was at 36 weeks post-menstrual age (PMA) or discharge home (whichever occurred first) in infants born at less than 32 weeks of gestation, and at day 56 of life in infants born with at least 32 weeks of gestation. Mild BPD was defined as breathing room air at that time point, moderate BPD as having supplemental oxygen, but need for less than 30% of oxygen and severe BPD as the need for more than 30% of oxygen at that time point. The standardized internal hospital guidelines were applied to determine the exact amount of oxygen needed.

Based on the definition of the clinical risk index for babies (CRIB: birthweight, gestational age, maximum and minimum fraction of inspired oxygen, maximum base excess during the first 12 h, and presence of congenital malformations) infants were given a CRIB score [26].

#### Term-born infants

Term-born healthy infants were antenatally recruited within a prospective birth cohort study since 1999 in the region of Bern, Switzerland [27]. Exclusion criteria for the study were preterm delivery (<37 weeks), significant perinatal disease including respiratory distress and later diagnosis of chronic respiratory disease (See Table 1 and 2 for patient characteristics).

**Table 1:** Anthropometric data of the study infants.

	Healthy		Bronchopulmonary dysplasia		
	Term	Preterm	mild	moderate	severe
Number of subjects	239	58	44	53	30
Gender (female/male)	110/129	24/34	14/30	20/33	13/17
Post-conceptional age at birth, wks	39.8+1.2 (37.0–42.3)	32.0+2.3 (27.1–36.7)	28.1+2.5 (23.9–35.4)	27.9+2.4 (24.3–36.1)	27.4+2.1 (24.4–33.6)
Prenatal smoke exposure^2^	31 out of 239	1 out of 52	8 out of 41	8 out of 45	3 out of 22
Birth weight, kg	3.40+0.45 (2.17–4.91)	1.66+0.57 (0.75–2.98)	1.10+0.42 (0.50–2.60)	1.02+0.40 (0.42–2.58)	0.87+0.29 (0.42–1.70)
Post-conceptional age at study date, weeks	44.8+1.3 (42–50)	44.3+2.1 (40–50)	44.3+2.3 (40–51)	44.8+2.5 (40–54)	44.8+3.2 (40–54)
Postnatal age at study date, days	35+5 (25–57)	85+21 (36–139)	113+24 (59–177)	118+26 (58–197)	121+26 (73–181)
Weight at study date, kg	4.4+0.6 (2.9–6.3)	4.1+0.7 (2.2–5.7)	3.9+0.7 (2.7–5.9)	3.8+0.7 (2.6–5.9)	3.7+0.8 (2.7–5.6)
Length at study date, cm	55+2.3 (48–62)	53+2.5 (47–58)	52+3.1 (46–59)	51+3.3 (45–60)	50+4.7 (43–62)
BMI^1^ at study date, kg/m^2^	14.5+1.2 (11–17)	14.3+2.7 (10–18)	14.5+1.5 (11–18)	14.5+1.7 (12–19)	14.3+1.5 (12–18)

Data is given as mean+/−SD (range).

1 Body mass index, calculated as weight divided by length.

2 Data on maternal smoking during pregnancy was available only in 399 out of the 424 infants.

**Table 2:** Clinical data of preterm infants.

	Healthy preterm	Bronchopulmonary dysplasia		
		mild	moderate	severe
Number of subjects	58	44	53	30
Prenatal steroids^1^	44 (76)	36 (86)	46 (88)	24 (86)
Chorioamnionitis^2^	9 (16)	25 (57)	20 (38)	11 (41)
Prenatal smoking	1 of 52 (2)	8 of 41 (20)	8 of 45 (18)	3 of 22 (14)
Endotracheal intubation	22 (38)	28 (64)	40 (75)	22 (73)
Continuous positive airway pressure (CPAP)	42 (72)	43 (98)	52 (98)	30 (100)
APGAR 5 min	8.4+1.2 (5–10)	7.7+1.4 (2–10)	6.8+1.7 (2–9)	7.4+2.1 (3–10)
APGAR 10 min	8.9+1.0 (5–10)	8.3+1.0 (5–10)	7.7+1.5 (3–9)	8.6+1.3 (6–10)
Days of supplementary oxygen^3^	8+7 (0–27)	46+13 (28–72)	78+29 (3–200)	144+108 (33–508)
CRIB Score^4^	1.4+1.8 (0–9)	4.1+3.1 (0–12)	5.3+4.0 (1–14)	6.0+3.3 (1–13)

Data is given as number (percentage) or mean+/−SD (range).

1 Prenatal steroids if given parenterally before delivery.

2 Diagnosis of maternal chorioamnionitis was made by postnatal histopathological examination of the placenta.

3 All infants were off oxygen at time of analysis.

4 CRIB Score is a postnatal score including birthweight, gestational age, minimum and maximum FiO_2_ and maximum Base Excess during the first 12 hours as well as malformations. It can range from minimum 0 points to maximum 23 [26].

### Lung function

Lung function measurements were performed via an infant face mask (Size 1; Homedica AG) during unsedated sleep in supine position with the head midline, according to ATS/ERS standards of infant lung function testing [28]. Measurements were performed following regular feeding of the infants, normally leading to natural sleep in this age group. After the infants fell asleep, the face mask was placed and the measurements were started. The first 20 to 30 breaths were rejected to allow adjustment of breathing pattern to the face mask. Then, 10 minutes of tidal breathing was recorded, followed by three measurements of multiple-breath washout. Flow was measured using an ultrasonic flowmeter (Spiroson®; EcoMedics AG, Dürnten, Switzerland). None of the infants were on inhaled pulmonary medication at the time of lung function measurements. Only data from behaviorally defined quiet sleep was used for analysis [29].

#### Multiple-breath washout

Lung volume and ventilation inhomogeneity were determined using multiple-breath washout (MBW) technique with 4% SF_6_ as previously described [30]. Technical acceptability was ascertained using recently-validated optimizations [31]. Outcomes were FRC at airway opening (FRC_ao_) and lung clearance index (LCI).

### Tidal breathing

For analysis, we used the first 100 regular breaths of tidal breathing during non-REM sleep from the total 10-min-recording, and excluded sighs and ten breaths before and after a sigh. From these, mean tidal breathing parameters of flow, volume and flow-volume loop were calculated as per the ERS/ATS standards for infant lung function testing [28]. Outcome parameters were tidal volume per body weight, respiratory rate, mean and peak tidal inspiratory and expiratory flow. To describe the shape of the tidal breathing flow-volume-loop we used the ratio of time to peak tidal expiratory flow (PTEF) and expiratory time (*t*_PTEF_/*t*_E_).

### Statistical analysis

#### Comparison between groups

Descriptive statistics and regression analysis were used to compare MBW and tidal breathing parameters between groups. These and subsequent analyses were performed using STATA 10 (STATA Corporation, College Station, TX, USA).

#### Discrimination between groups

To investigate which lung function parameters discriminate best between groups, receiver-operator characteristics (ROC) curves were calculated. First, we grouped healthy term and preterm infants together and compared them with BPD infants; second within the healthy infants we compared term-born with preterm infants.

#### Clinical determinants of lung function in preterm infants

We also assessed which clinical variables may determine lung function in preterm infants. We performed univariable linear regression analyses with the following main lung function parameters as outcomes: FRC_ao_, LCI, respiratory rate, tidal volume and *t*_PTEF_/*t*_E_. We first entered the following clinical variables as explanatory variables into the univariable model: weight and length (both determined at the time of the study), post-conceptional age, gender, prenatal steroids, maternal smoking during pregnancy, maternal chorioamnionitis, days of supplementary oxygen, duration of intubation, duration of continuous positive airway pressure (CPAP), surfactant medication, postnatal weight gain and CRIB score. We then performed multivariable linear regression analysis for each of the outcome parameters and included all explanatory variables that were significantly associated (p-value<0.05) in the univariable analysis. We then used a backward stepwise exclusion strategy by dropping the variable with the highest p-value until only significantly associated variables were left and built one final model including all the variables that were significantly associated (p-value<0.05) with any of the outcome parameters. This final model is reported for all the five lung function parameters.

## Results

Between 1999 and 2007, the study enrolled 185 preterm and 239 term-born infants; anthropometric data are given in Table 1 and 2. Data from 173 (94%) preterm and 228 (95%) term-born infants were used for tidal breathing analysis and from 146 (79%) and 181 (76%) for MBW analysis. Reasons for exclusion were insufficient duration of quiet sleep (12 preterm and 11 term-born infants), lower respiratory tract infection before the measurement (3 term-born infants) and strict quality control criteria of MBW (27 preterm and 44 term-born infants).

### Comparison of lung function between groups

#### Multiple-breath washout

**Figure 1:**
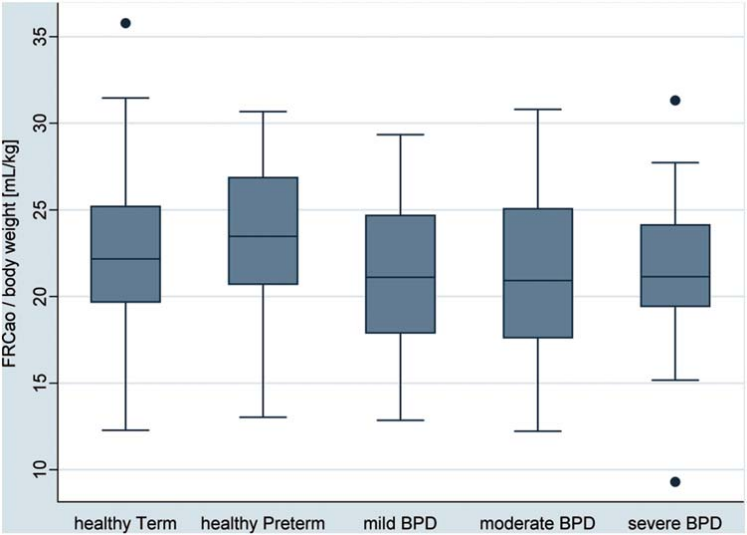
Box plots of the respective lung function values by subject groups. Subjects were grouped according to post-conceptional age at birth (term-born and preterm) and according to disease state based on ATS definition of BPD (healthy preterm, mild, moderate and severe BPD) [6]. The boxes indicate the median and the 25^th^ and 75^th^ percentile, the whiskers the upper and lower adjacent values. Outside values are shown as separate dots. Box plots are shown for FRC at airway opening per weight in mL/kg.

There was no clear trend in changes of FRC adjusted for body weight with increasing disease severity (Figure 1, Table 3). However, both healthy term-born (mean 22.6 mL/kg, 95-% CI 15.9–29.8 mL/kg) and healthy preterm infants (23.4 mL/kg, 95-% CI 15.9–29.5 mL/kg) had slightly higher FRC per body weight than the BPD infants combined (21.4 mL/kg, 95-% CI 13.8–25.4 mL/kg; p-value of 0.027 and 0.017, respectively). Also of note was the wide range in FRC values in all groups, with great overlap between groups.

**Table 3:** Comparison of lung function parameters between groups.

	Healthy		Bronchopulmonary dysplasia		
	term	preterm	mild	moderate	severe
**Multiple-breath washout**					
	22.6+4.2	23.4+4.4	21.4+4.2	21.4+5.0	21.3+4.4
	(12.3–35.8)	(13.0–30.7)	(12.9–29.3)	(12.2–30.8)	(9.3–31.3)
FRC_ao_, mL/kg	NA	0.279	0.164	0.106	0.176
	7.0+0.8	6.9+0.7	7.0+0.7	6.9+0.7	7.2+1.0
	(5.5–10.1)	(5.2–8.5)	(6.0–8.8)	(5.9–8.7)	(5.9–9.4)
LCI	NA	0.661	0.860	0.716	0.191
**Tidal breathing parameters**					
	7.5+1.4	7.6+1.1	7.5+1.9	7.5+2.3	7.3+1.4
	(4.3–11.1)	(5.3–10.1)	(5.5–17.4)	(4.7–19)	(4.8–10.8)
Tidal Volume, mL/kg	NA	0.690	0.950	0.789	0.551
	45+11	48+10	50+9	53+14	58+17
	(24–79)	(29–83)	(36–69)	(31–95)	(33–103)
Respiratory rate, per minute	NA	0.114	0.016	<0.001	<0.001
	328+65	359+62	365+75	381+93	404+84
	(179–612)	(238–513)	(231–660)	(259–796)	(272–569)
Minute Ventilation, mL/kg*min	NA	0.006	0.002	<0.001	<0.001
	43+10	44+10	43+11	44+12	43+9
	(20–80)	(24–62)	(18–75)	(23–76)	(25–59)
Mean expiratory flow, mL/s	NA	0.741	0.850	0.706	0.968
	54+9	55+12	54+12	55+13	56+11
	(30–82)	(33–82)	(30–90)	(34–92)	(32–76)
Mean inspiratory flow, mL/s	NA	0.825	0.703	0.733	0.515
	64+14	66+16	64+16	71+21	74+15
	(26–111)	(39–110)	(26–98)	(40–134)	(34–96)
Peak expiratory flow, mL/s	NA	0.321	0.899	0.002	0.001
	77+13	76+17	73+16	76+18	78+15
	(42–113)	(42–113)	(44–121)	(50–124)	(42–108)
Peak inspiratory flow, mL/s	NA	0.876	0.189	0.815	0.734
	36+11	31+9	28+7	26+9	23+7
	(15–73)	(14–59)	(15–40)	(11–62)	(12–38)
*t*_PTEF_/*t*_E_, in %	NA	0.001	<0.001	<0.001	<0.001

Data is given as mean+SD, (range) and p-value as determined by an unpaired t-test compared to the healthy term born group.

**Figure 2:**
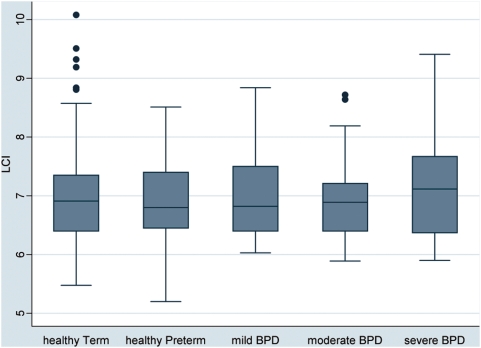
Box plots of the respective lung function values by subject groups. Subjects were grouped according to post-conceptional age at birth (term-born and preterm) and according to disease state based on ATS definition of BPD (healthy preterm, mild, moderate and severe BPD) [6]. The boxes indicate the median and the 25^th^ and 75^th^ percentile, the whiskers the upper and lower adjacent values. Outside values are shown as separate dots. Box plots are shown for lung clearance index (LCI).

LCI did not differ systematically between groups (Figure 2, Table 3).

#### Tidal breathing parameters

Tidal volume per body weight was also comparable between groups (Table 3), respiratory rate increased with increasing disease severity (Table 3; p-value for trend <0.001), leading to an increased minute ventilation and increased peak expiratory flows with disease severity (Table 3). No differences between groups were found for mean tidal expiratory and inspiratory flows (Table 3).

**Figure 3:**
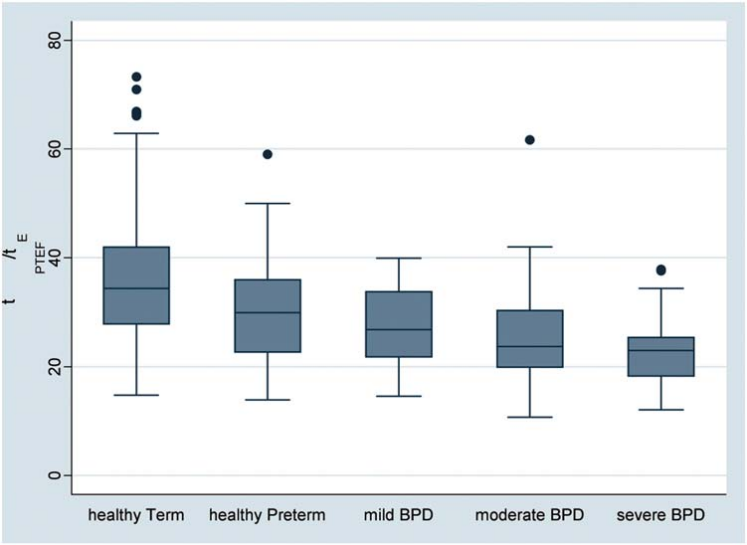
Box plots of the respective lung function values by subject groups. Subjects were grouped according to post-conceptional age at birth (term-born and preterm) and according to disease state based on ATS definition of BPD (healthy preterm, mild, moderate and severe BPD) [6]. The boxes indicate the median and the 25^th^ and 75^th^ percentile, the whiskers the upper and lower adjacent values. Outside values are shown as separate dots. Box plots are shown for *t*_PTEF_/*t*_E_. The p-value for trend obtained by regression analysis was <0.001.

A significant trend was found for changes between groups for *t*_PTEF_/*t*_E_ (Figure 3), which decreased with disease severity (p<0.001, Table 3). Also of note was the decreasing range in *t*_PTEF_/*t*_E_ with disease severity.

### Discrimination between groups

**Figure 4:**
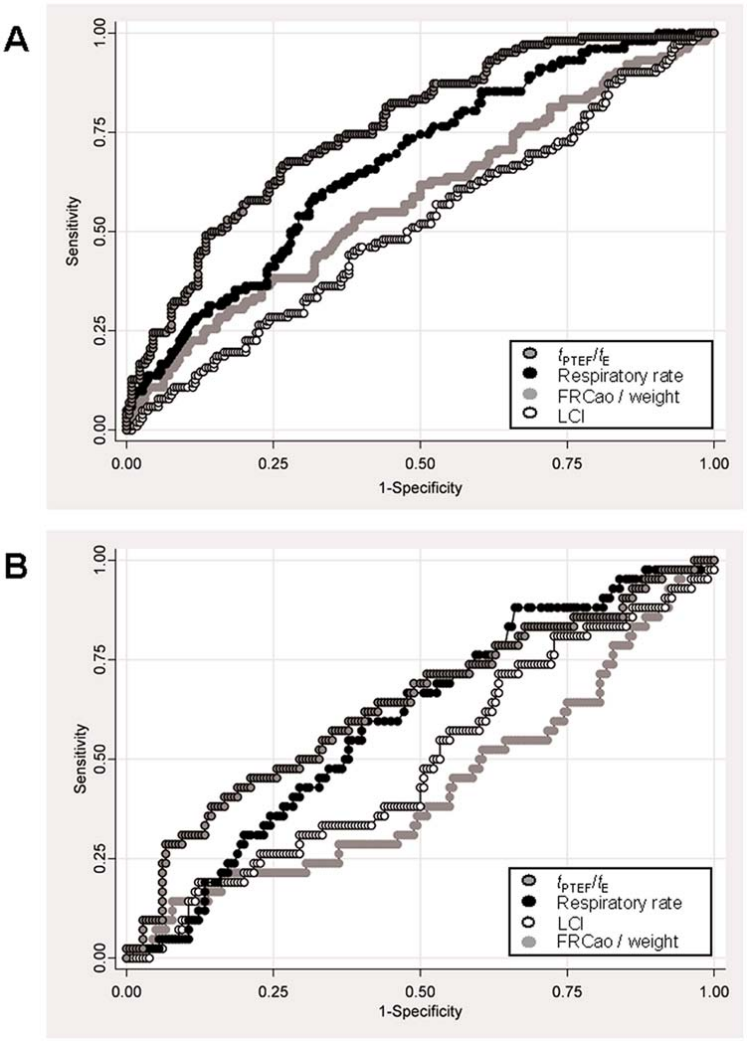
Receiver-operator characteristic (ROC) curves comparing *t*_PTEF_/*t*_E_ (dark gray symbols with black outline), respiratory rate (black symbols), FRC per weight (light gray symbols without outline) and LCI (white symbols) between groups. a) ROC curve comparing the ability of above mentioned lung function parameters to discriminate between healthy infants (term-born and preterm) and BPD infants using data of 221 healthy and 103 preterm infants. The resulting area under the curve is 0.58 for FRC_ao_, 0.52 for LCI, 0.67 for respiratory rate and 0.76 for *t*_PTEF_/*t*_E_. b) ROC curve comparing the ability of above mentioned lung function parameters to discriminate between 179 term-born and 43 preterm healthy infants; BPD infants were not considered for this analysis. The resulting area under the curve is 0.43 for FRC_ao_, 0.50 for LCI, 0.60 for respiratory rate and 0.64 for *t*_PTEF_/*t*_E_.

ROC curve analysis showed that *t*_PTEF_/*t*_E_ and respiratory rate both discriminate better between healthy and BPD infants than FRC and LCI (Figure 4a). When comparing term-born and preterm-born healthy infants, these parameters remained the best discriminating factors, but the discrimination between term-born and preterm was not as good as between health and disease (Figure 4b), indicating that these parameters are more an expression of functional abnormalities of BPD rather than prematurity itself.

### Clinical determinants of lung function in preterm infants

We performed uni- and multivariable linear regression analysis in order to assess the association between clinical variables reflecting severity of lung disease and lung function values in preterm infants. Table 4 shows the final model, which includes all variables that were significantly or with borderline significance (days of CPAP) associated with any of the main outcome parameters in multivariable analysis. We used the same model for all different lung function outcomes. Taken together, parameters of lung size such as FRC and tidal volume were independently related to body size (weight and/or length). *T*_PTEF_/*t*_E_ showed the strongest association with duration of supplementary oxygen. Of note were the varying adjusted R^2^ values, which describe the goodness of fit of the statistical model. These values were between 3% for LCI and 39% for tidal volume, indicating that e.g. 3% of the variability in LCI compared to 39% of the variability in tidal volume can be explained by all the factors in the model.

**Table 4:** Association of clinical variables and lung function parameters in 185 preterm infants.

	Univariable model			Multivariable model^1^^,^ 2		
	coefficient	CI 95%	p-value	coefficient	CI 95%	p-value
FRC_ao_, mL^3^						
Weight	13.9	10.2–17.7	<0.001	6.6	0.3–12.9	0.040
Length	3.2	2.4–4.0	<0.001	1.6	0.2–3.0	0.023
Post-conceptional age	2.0	1.0–3.1	<0.001	1.0	−0.4–2.4	0.154
Days of Oxygen	−0.08	−0.13–−0.04	<0.001	−0.04	−0.09–0.02	0.192
Days of CPAP	−0.24	−0.40–−0.09	0.002	0.002	−0.2–0.2	0.984
LCI^3^						
Weight	−0.18	−0.34–−0.02	0.026	−0.05	−0.34–0.23	0.704
Length	−0.04	−0.08–−0.01	0.020	−0.03	−0.09–0.04	0.402
Post-conceptional age	−0.01	−0.05–0.03	0.627	0.03	−0.04–0.09	0.429
Days of Oxygen	0.002	0.0002–0.004	0.024	0.002	−0.00003–0.005	0.053
Days of CPAP	0.002	−0.003–0.008	0.426	−0.00004	−0.009–0.009	0.993
Tidal volume, mL^3^						
Weight	7.1	5.7–8.4	<0.001	5.7	3.3–8.0	<0.001
Length	1.4	1.1–1.7	<0.001	0.3	−0.19–0.86	0.214
Post-conceptional age	0.5	0.08–0.90	0.021	−0.07	−0.6–0.5	0.799
Days of Oxygen	−0.02	−0.04–−0.002	0.029	0.006	−0.02–0.03	0.611
Days of CPAP	−0.09	−0.15–−0.03	0.002	−0.06	−0.14–0.01	0.102
Respiratory rate, /min^3^						
Weight	−4.6	−7.0–−2.1	<0.001	1.2	−3.0–5.4	0.575
Length	−1.2	−1.8–−0.7	<0.001	−1.3	−2.2–−0.36	0.007
Post-conceptional age	−0.6	−1.2–0.06	0.076	0.8	−0.8–1.75	0.074
Days of Oxygen	0.05	0.02–0.08	0.001	0.04	0.002–0.08	0.039
Days of CPAP	0.15	0.06–0.24	0.002	0.13	−0.003–0.27	0.055
*t*_PTEF_/*t*_E_ 3						
Weight	−0.9	−2.7–0.8	0.295	−3.8	−6.6–0.9	0.010
Length	0.07	−0.3–0.5	0.741	0.5	−0.13–1.14	0.120
Post-conceptional age	0.83	0.42–1.24	<0.001	0.7	0.08–1.33	0.028
Days of Oxygen	−0.04	−0.06–−0.02	<0.001	−0.04	−0.06–−0.01	0.007
Days of CPAP	−0.09	−0.15–−0.03	0.004	0.03	−0.06–0.13	0.483

1 The table gives all clinical parameters (exposure) that showed a significant association (p-value<0.05) with the any of the respective lung function values (outcome) adjusted for all other variables in the model, as shown in the table.

Results were unchanged if further adjusted for maternal smoking during pregnancy, a factor known to influence both lung function and control of breathing. Due to low numbers, in our cohort, maternal smoking during pregnancy alone was not associated with any of the lung function outcomes.

2 The adjusted R-square values, indicating how much of the variability of the outcome is explained by the parameters in the model for the respective lung function parameters are as follows: FRC 34%; LCI 3%; Tidal volume 39%; Respiratory rate 15% and *t*_PTEF_/*t*_E_ 14%.

3 The change of units of the respective lung function parameter by the following change of the clinical variables is given: per kg higher body weight at the time of the study; per cm higher body length at the time of the study; per week post-conceptional age; per day of supplementary oxygen; per day of CPAP.

### Variability of lung volume

In order to explain the lack of trend in tidal volume and FRC with BPD severity, and the wide range in tidal volume and FRC observed, in a post-hoc approach we examined how potential factors determining lung volumes would differ with prematurity and disease severity. We hypothesized that FRC is determined by three components: body size, disease status (prematurity and BPD) and neuro-respiratory compensatory mechanisms. The last component is likely to be dominant in influencing short-term variability/adaptability of FRC. To determine this influence, we examined the association between weight (as a surrogate for body size) as the explanatory variable and lung and tidal volumes as outcomes, since weight was the strongest contributor to volume (Table 4). The R^2^ value of the regression model gives a measure of how much the variability in the outcome is determined by the exposure. An R^2^ value of 1 or 100% would mean lung volume is only determined by weight and there is no contribution of potential neuro-respiratory mechanisms to variability in lung volume. An R^2^ value of 0% would mean that lung volume is not determined at all by weight, but that other factors contribute largely to the variability in lung volume. Examining these associations separately first by prematurity and second by BPD status allowed us to determine how the contribution of these mechanisms differs with disease, which may set mechanical limits to the neuro-respiratory control.

**Figure 5:**
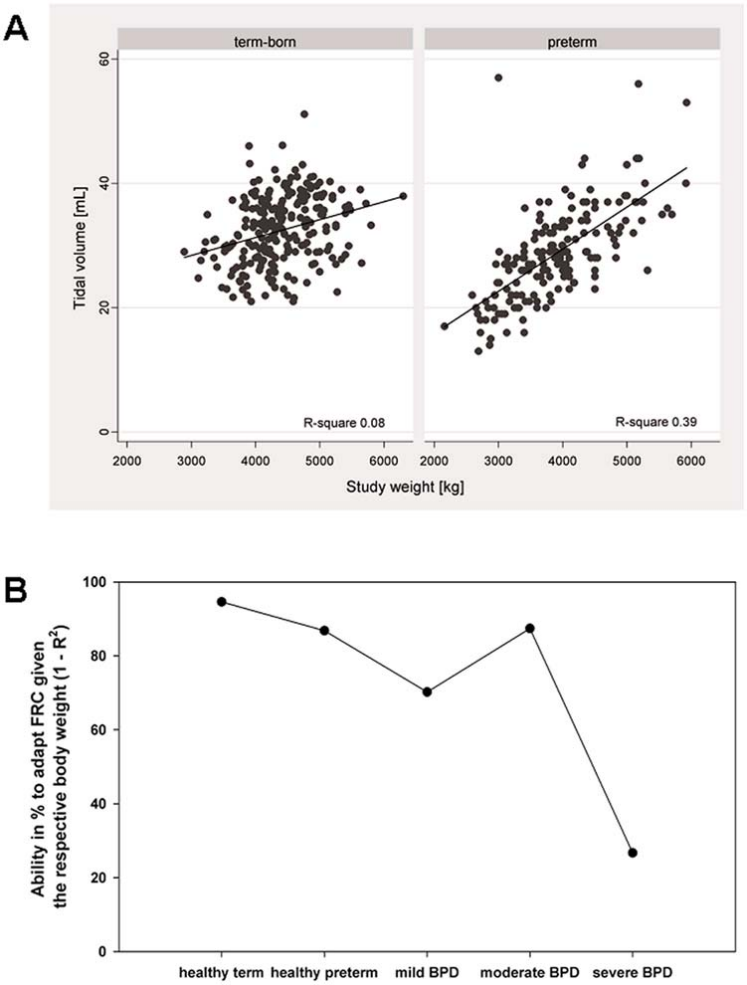
a) Tidal volume versus weight for term and preterm infants. The black line indicates the regression line with a coefficient of correlation of 0.31 for the term-born infants and a coefficient of correlation of 0.65 for the preterm infants and the respective R^2^ values given in the figure. b) Variability in lung volume determined by weight depending on disease severity. The graph shows 1−R^2^ value of the regression model between weight and FRC. The R^2^ value indicates how much of the variability in lung volume can be explained by the weight, and thus the 1−R^2^ is a measure of how much infants are able to change their lung volumes given their body size.

We found a much tighter correlation for preterm compared to term-born infants between weight and tidal volume (R^2^ value of 8% for term-born and of 39% for preterm infants; see figure 5a) as well as between weight and FRC (R^2^ value of 5% for term-born and of 27% for preterm infants). Figure 5b shows that the R^2^ tended to increase with disease severity, i.e. the adaptability of FRC given the respective weight (reflected by 1−R^2^) is decreased, or in other words, with increasing disease severity lung volume is increasingly determined by body size. This could mean that in the BPD infants neuro-respiratory mechanisms are either already activated or mechanical properties limit the activation of these mechanisms. Comparable results were obtained for tidal volume. None of the associations improved by additionally including other variables, such as e.g. length or duration of oxygen.

## Discussion

### Summary

Using lung function tests performed in accordance to recently-published recommendations, we showed in a large cohort that end-expiratory lung volume and ventilation inhomogeneity measured during natural sleep is comparable in term-born and preterm infants and in infants with different stages of BPD, when adequately adjusted for body weight. Tidal breathing parameters changed with increasing BPD severity, discriminated best between health and BPD, and showed the strongest association with duration of supplementary oxygen. In a post-hoc analysis, a tighter correlation was found between lung volumes and weight in preterm infants than in health, with a trend seen for increasing disease severity.

### Comparison with other studies

Caution should be exercised in comparing these results with other studies. Apart from the differences we have highlighted, most previous studies included infants with the “old” form of BPD, with different pathological causes possibly leading to a different functional pattern of their lung disease [2], [4].

#### FRC and LCI

In contrast to our study, Hjalmarson and Sandberg showed distinct differences in FRC and LCI between preterm and term-born infants [12] as well as between BPD and non-BPD infants [11]. However, they used 100% oxygen as washout gas, known to induce atelectasis [22] and influence breathing pattern – infants with BPD show a blunted response to hyperoxia, thus 100% oxygen would lead to a smaller decrease in tidal volumes with consequently less rise in end-expiratory volume than in healthy infants [32]. In addition, this group studied healthy infants within 28 to 72 h after birth [12], where adaptation to postnatal breathing pattern is not fully established and response to hyperoxia is different than later in life [33]. Another group using SF_6_ and equipment comparable to ours in a smaller group of infants obtained similar results as we did, with no differences in FRC adjusted for body weight between BPD infants, preterm and term-born controls [17].

#### Tidal breathing parameters

Few studies have examined tidal breathing parameters in greater detail. Clarke et al. report similar results as we do, with *t*_PTEF_/*t*_E_ being much smaller in infants with severe BPD compared to healthy controls measured at the same age [34]. Another study used ROC curves to compare BPD and non-BPD infants, and found that inspiratory time discriminated best between BPD and healthy infants, much better than *t*_PTEF_/*t*_E_ [35]. Besides the fact that sedation was used in eleven out of 48 infants in the BPD group compared with only one out of 48 healthy infants, we do not have any obvious explanation for this discrepant finding.

### Possible mechanisms

Overall, if appropriately adjusted for body weight and measured at the same post-conceptional age, FRC in BPD infants is comparable to FRC in healthy infants and thus higher than expected. We hypothesize that active mechanisms are involved in elevating FRC in BPD infants. This can be done by early inspiratory muscle activity or braking of the expiratory flow [36]. Comparable mean and peak expiratory flows between groups make braking in BPD infants unlikely, suggesting that during unsedated sleep they use their respiratory muscle activity to dynamically maintain end-expiratory lung volume. The large range of FRC values in the whole group of infants also supports this idea. Perhaps in this age group there is no “normal” FRC, but end-expiratory level is determined by weight (which cannot change quickly) as well as by dynamically changing muscle activity. In contrast to term-born infants, preterm infants breathe within a much narrower range around their expected lung volume. We hypothesize that with increasing disease severity, BPD infants are at the limits of their breathing pattern adaptability in order to maintain end-expiratory lung volume, and thus these infants have to breathe with greater physiological constraints.

In agreement with this argument we found an increased respiratory rate with disease severity in combination with comparable tidal volumes between BPD and healthy infants. Because of an increased ventilatory need, BPD infants show a higher minute ventilation than healthy infants. We hypothesize that due to the sigmoid shape of the compliance curve, it may be energetically more efficient for these infants to increase minute ventilation by elevating respiratory rate while maintaining tidal volume.

The decreased *t*_PTEF_/*t*_E_ in BPD may in this case be suggestive of a lower compliance with increasing disease severity, in accordance with recent studies showing decreased flows during forced expiration in sedated infants [16]. It has to be emphasized, that *t*_PTEF_/*t*_E_ reflects the neuromuscular response to respiratory mechanics and is therefore determined by the importance of establishing a stable FRC based on the underlying mechanical properties of the respiratory system and the interdependence between respiratory timing, modulation of expiratory flow and dynamic elevation of lung volume [37], [38]. Without additional measurements of total lung volume and resistance or compliance the exact influence of any of these factors cannot be disentangled. Another potential source of variability lies in the fact that merely placing a mask and adding additional dead space is known to alter the breathing pattern [39], possibly in different ways in health and disease.

The finding of normal LCI in BPD infants may be due to methodological reasons. Although no studies on the effect on LCI exist, SF_6_ has a lower viscosity and lower diffusibility than helium and may not be sensitive enough to detect small changes. Structural considerations may also play a role. Lungs of BPD infants are known to be developmentally less complex with fewer generations of acini. Thus it might be that this simpler and more homogeneous lung structure in the BPD infants would lead to lower “baseline” LCI and that the normal LCI measured in these infants in fact indicate increased ventilation inhomogeneity. Alternatively, normal LCI in these infants may also reflect the milder degree of “new” BPD with more peripheral location of airway involvement and airway closure leading to more regional than overall changes in ventilation distribution [40].

### Methodological strengths and limitations

To our knowledge, this is the first study systematically assessing tidal breathing parameters, lung volume and ventilation inhomogeneity in a cohort of preterm infants with different stages of “new” BPD, in comparison to age-matched healthy term born infants. Age-matching has been an ongoing subject of debate regarding possible influence on findings in previous studies [41], and is not only important due to differences in lung size, but also in maturation of neural regulatory mechanisms [9]. In this regard it is important to mention that subjects showed proportionate growth as indicated by comparable body mass indices between groups. In addition, preterm and term-born infants were recruited during the same time period and within the same area. Lung function measurements were done using the same equipment and measurement procedure throughout the whole study period based on ATS/ERS standards [25], with washout done without the use of 100% oxygen based on latest technical optimizations [31]. We analyzed 100 breaths for tidal breathing parameters, giving more robust estimates than 30 breaths as recommended by the standards [28].

We studied infants during unsedated sleep. Compared to studies during sedation, this reflects much better the natural state of breathing. Moreover, only in unsedated infants a possible dynamic regulation of end-expiratory level by adapting breathing pattern can be studied [36], [42].

Clearly the use of a clinical definition of oxygen need for defining BPD severity comprises a certain risk of misclassification. However, this definition is relevant to daily practice. Moreover the decreasing and clear trend seen in some of our results, suggests that this classification indeed is associated with differences in lung function. Misclassification of disease severity, if it had occurred, would have instead blurred the lung function differences observed between groups.

We are unable to confirm the involvement of active mechanisms by which lung volume is maintained as we did not measure resistance, compliance, or control of breathing parameters. Furthermore, due to the observational nature of our data, causal relationships cannot be deduced from our findings. In contrast to physical factors such as weight, clinical factors (such as duration of oxygen) may be a proxy for disease severity and thus may also be a consequence of changes in lung development rather than a cause.

### Relevance

Studies showing increased respiratory morbidity and decreased lung function in childhood and adolescent survivors of BPD suggest that late pulmonary sequelae of BPD leads to increasing incidence of chronic obstructive pulmonary disease in future years [43]–[47]. Thus it is important to understand early changes of the physiological development in these children. Our findings suggest that during natural sleep without distress, even preterm infants with severe forms of BPD are capable of maintaining their end-expiratory lung volume. This active maintenance of lung volume has implications for understanding clinical situations in premature infants. Due to their limited reserve and limitations in dynamic variability even with minimal overt respiratory signs, this capacity may change during phases of distress. For example, changes in the control of breathing (sedation or immaturity) or further mechanistic disturbance (respiratory infections) may lead to decompensation of the system, since their active breathing control mechanisms may be disturbed or abruptly reach the limits.

We found decreasing *t*_PTEF_/*t*_E_ values with increasing BPD severity. In addition to its association with dynamic regulation of lung mechanics as discussed above, a decreased *t*_PTEF_/*t*_E_ may have long-term consequences for BPD infants. At the 10-year follow-up of 616 children from a birth cohort, Haland et al. were not only able to demonstrate that *t*_PTEF_/*t*_E_ after birth correlated with mid-expiratory flow rate at 10 years, but they could also show that infants with a *t*_PTEF_/*t*_E_ below the median have a 2.1-fold risk of having current asthma at 10 years [48]. Thus, changes in lung mechanics already existent at birth may be the reason for the higher prevalence of asthmatic disease in young adults born prematurely, even though atopic diseases occur less often in these subjects [49].

### Conclusion

In conclusion, parameters describing the shape of the tidal breathing flow-volume loop seem to be simple surrogate markers of breathing alterations in infants with BPD, which are easy to obtain and thus seem to be useful in the clinical setting. Our findings are more representative of the normal state of breathing in these infants, but consistent with findings of reduced forced flows in sedated infants. Normal end-expiratory lung volumes during natural sleep in BPD infants suggests that they have a high capacity to compensate by dynamically adapting their breathing pattern, which they are able to do when neither sedated nor clinically stressed. These findings are in agreement with the clinical picture, and have important implications for understanding the pathophysiology and management of the more recent form of BPD.
